# Iliac-enteric fistula managed by endovascular covered stent placement using extra-vascular microwire snaring: a case report

**DOI:** 10.1186/s42155-020-00109-2

**Published:** 2020-03-23

**Authors:** Amgad M. Moussa, Constantinos T. Sofocleous

**Affiliations:** Department of Interventional Radiology, Memorial Sloan Kettering Cancer Center, 1275 York Avenue, Suite H-188, New York, NY 10065 USA

**Keywords:** Snare, Extra-vascular, Pseudoaneurysm, Covered stent, Iliac-enteric fistula

## Abstract

**Background:**

Although snaring technique is a commonly used tool in the interventional radiologists’ armamentarium, there are no reports of its use in an extra-vascular space to achieve access across a pseudoaneurysm that was otherwise non-traversable.

**Case presentation:**

We describe a case of an iliac-enteric fistula between a ruptured pseudoaneurysm of the external iliac artery and a surrounding contained colonic perforation, where access across the pseudoaneurysm was achieved only after snaring of the microwire from within the contained colonic perforation and back into the intra-vascular space, allowing the placement of a covered stent and control of the bleeding.

**Conclusions:**

The described technique may be useful in situations where other, more conventional, endovascular techniques fail to achieve access across the bleeding pseudoaneurysm. While it was life-saving in this case, this technique should only be used in very limited scenarios, specifically in the palliative setting and when surgical management is not an option.

## Background

The use of snaring technique is a relatively common problem-solving tool used by interventional radiologists in a variety of different clinical scenarios. The most common uses include retrieval of intra-vascular foreign bodies (such as Inferior vena cava filters, fractured catheter fragments and stents) and achieving access in challenging vascular anatomy in specific scenarios (Iliescu and Haskal 2012; Woodhouse and Uberoi 2013; Hanaoka et al. 2006). During complex interventional procedures or following intra-procedural complications, it is usually the last resort before surgical intervention (Palmer et al. 2019).

We describe a case of unorthodox use of the snaring technique to achieve access across an iliac-enteric fistula between a ruptured pseudo-aneurysm of the external iliac artery and a contained perforation of the sigmoid colon, presenting with massive lower gastrointestinal bleeding. The snare technique was used within the contained colonic perforation to achieve access across the ruptured pseudoaneurysm, after attempts to cross the pseudoaneurysm from antegrade and retrograde approaches failed due to severe stenosis of the vessel, due to impingement by surgical clips and prior pelvic radiation. Achieving access across the pseudoaneurysm allowed placement of covered stents and exclusion of the pseudoaneurysm, with control of the bleeding.

While the conditions for placement of a covered stent were far from optimal, owing to the infected field where the stent will be placed as well as the snaring process taking place within the colonic perforation, the emergent nature of the case and the lack of immediate surgical options for the patient (patient has repeatedly refused surgery for colonic perforation) supported this decision. In addition, reported experience from emergent endovascular management of aorto-duodenal fistulas, which can be considered an infected field, supports the use of covered stents, along with the use of long-term antibiotics to potentially prevent infection of the endovascular stent graft (Morikawa et al. 2017).

## Case presentation

**Fig. 1:**
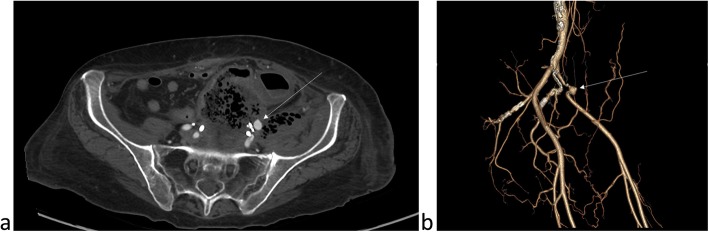
An **a** axial cut of a Computed Tomography Angiogram and **b** a 3D reconstruction showing the pseudoaneurysm (arrow) arising from the left external iliac artery with narrowing of the vessel proximal and distal to the pseudoaneurysm appreciated on the 3D reconstruction, which are related to surgical clips

A 64-year-old woman with recurrent metastatic endocervical cancer and history of pelvic surgery and radiation, with recent history of contained sigmoid colon perforation (managed conservatively after patient refused surgery) presented to the emergency department with recurrent attacks of bright red blood per rectum over the past day (10 attacks) with dizziness, tachycardia and a Hb of 6.6 mg/dL. Patient has indwelling ureteric stents that were exchanged 6 weeks prior, with no complications. Computed Tomography Angiography of the abdomen and pelvis showed a 9 mm pseudoaneurysm arising from the proximal left external iliac artery related to impingement on the vessel by two surgical clips, and in close proximity to the contained sigmoid perforation, which was expected to be the source of the hematochezia (Fig. 1a & b). Patient was taken to the interventional radiology angiography suite for endovascular management of the pseudoaneurysm.

**Fig. 2:**
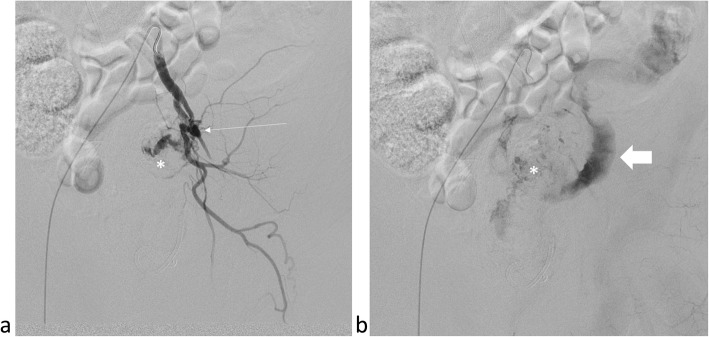
An **a** arterial phase and **b** delayed phase angiogram of the left Common iliac artery showing the pseudoaneurysm (thin arrow) with noted contrast extravasation into the contained colonic perforation (asterisk) with the sigmoid colon (thick arrow) seen on the delayed phase image

**Fig. 3:**
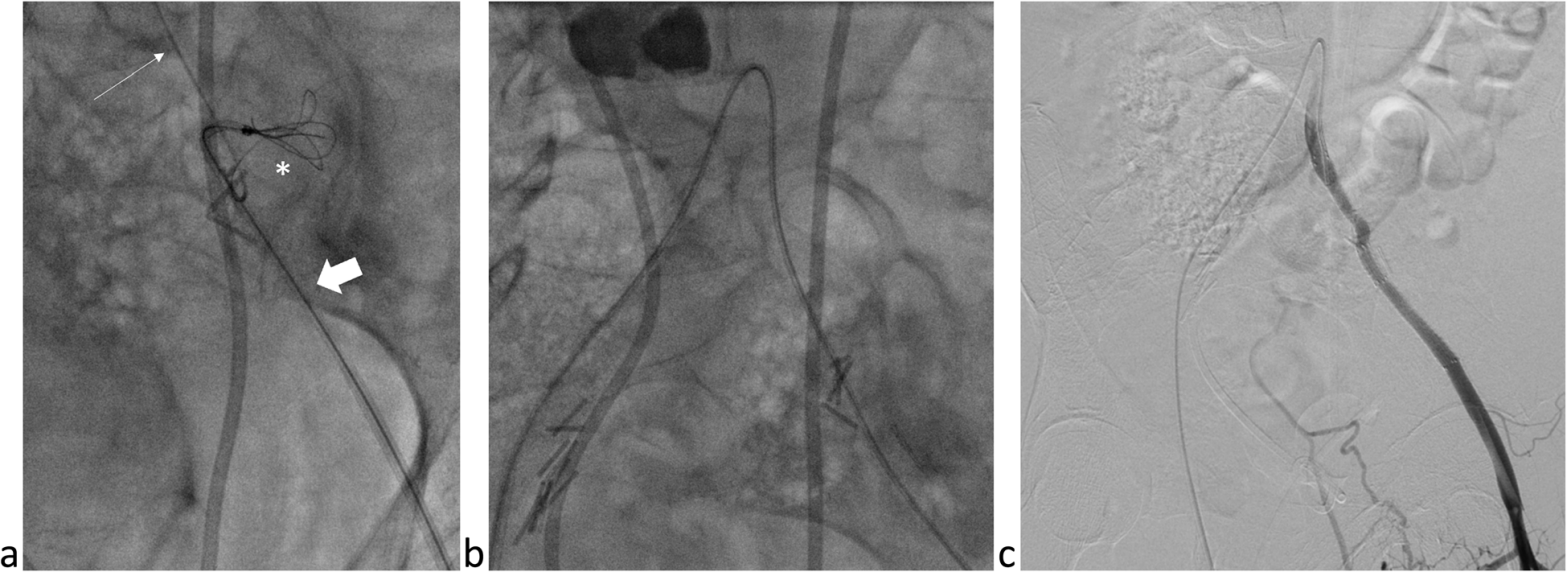
A Fluoroscopic image **a** showing the microcatheter and microwire from the right sided arterial access (thin arrow) and the snare from the left sided arterial access (thick arrow) together within the extra-vascular space (asterisk) followed by successful snaring and through-and-through access **b** across the pseudoaneurysm. An arterial phase angiogram **c** of the left external iliac artery following deployment of the covered stents shows exclusion of the pseudoaneurysm with no contrast extravasation

In the angio suite, the patient had another attack of bright red blood per rectum immediately before starting the procedure. Right common femoral artery access was achieved and a Sos Omni 2 catheter (Soft-Vu; Angiodynamics Inc., New York, USA) was used to cross into the left Common Iliac artery. Left Common Iliac artery angiography showed the pseudoaneurysm arising just distal to the origin of the left external iliac artery, with stenosis and tortuosity just proximal to its origin, likely related to the surgical clips (Fig. 2a), with contrast extravasation seen reaching the contained colonic perforation, and delayed images showing contrast reaching the sigmoid colon (Fig. 2b). Attempts to cross the pseudoaneurysm using a 2.8 Fr microcatheter (Progreat; Terumo Medical, Tokyo, Japan) were unsuccessful, likely due to severe stenosis by the surgical clips, with passage of the microcatheter into the colonic perforation. Left common femoral artery access was achieved and attempts to cross the pseudoaneurysm retrograde using a 2.8 Fr microcatheter were also unsuccessful, with the microcatheter again leaving the intra-vascular space and passing into the colonic perforation cavity. The right access microcatheter was then navigated into the colonic perforation cavity over a 0.018″ inch microwire (Glidewire GT guidewire; Terumo Medical, Tokyo, Japan) and a snare device (ENsnare 9–15 mm; Merit Medical, Philadelphia, USA) was then navigated into the same cavity from the left access (Fig. 3a). The microwire was snared successfully within the colonic perforation cavity and pulled out of the left groin access, achieving access across the pseudoaneurysm (Fig. 3b). The microcatheter was then advanced across the pseudoaneurysm and out of the left groin access. With the distal tip of the microcatheter secured outside, the 0.018″ inch Glidewire was exchanged for an exchange-length stiff microwire (300 cm V-18 Controlwire; Boston Scientific, Marlborough, Massachusetts) which was secured outside the patient’s body, providing secure access across the pseudoaneurysm. The microcatheter was removed, and two overlapping 7 mm × 5 cm covered stents (Viabahn; Gore medical, Delaware, USA) were deployed over the wire across the pseudoaneurysm. The stent diameter was chosen based on measurements of the proximal external iliac artery, and initially one stent was placed covering the pseudoaneurysm, but the stent was extended distally when angiographic evidence of dissection was seen distal to the stent. The final angiogram showed no contrast extravasation with good flow into the left External Iliac Artery (Fig. 3c).

The patient was placed on broad-spectrum antibiotics immediately following the procedure and admitted to the intensive care unit. Five days later, the patient was discharged from the intensive care unit after stabilization of her acute clinical course, with negative blood cultures. Following extensive family discussions and given progression of her disease, the patient requested to assume Do Not Resuscitate/Do Not Intubate status and the patient succumbed to her disease 20 days later.

## Discussion and conclusions

While the use of snaring technique is relatively common among interventional radiologists, we were not able to find in the literature any cases where a snaring device was used to retrieve an extra-vascular device back into the vascular tree. One similar reported case, though not identical, describes the use of two snare devices and a Chiba needle to achieve extra-vascular recanalization in a patient with chronic total occlusion of the popliteal artery (Kahn and Kaufman 2016).

Covered stent placement for exclusion of pseudoaneurysms from the circulation is gaining popularity, owing to the ability to maintain vessel patency and selectively treat the pseudoaneurysm, as opposed to other endovascular techniques (e.g. coil embolization of the parent artery proximal and distal to the pseudoaneurysm) (Madhusudhan et al. 2016; Kim et al. 2014). Deployment of covered stents across a pseudoaneurysm, however, requires wire access across the pseudoaneurysm, which is often achievable using antegrade or retrograde passage of a guidewire across the pseudoaneurysm. In this patient, these methods failed in achieving access across the pseudoaneurysm, owing to the vessel stenosis caused by the surgical clips, along with the rupture of the pseudoaneurysm, which prevented navigation of a wire across it. Employment of the described technique allowed wire access across the pseudoaneurysm. In addition, sacrifice of the vessel by coil embolization proximal and distal to the pseudoaneurysm was not an option because while evidence of chronic stenosis of the External iliac artery is seen on imaging, collateral vessels reaching the common femoral artery that would allow doing that without causing lower limb ischemia were not seen.

The use of this technique, though with expected grim consequences (e.g. sepsis) due to direct contamination of the bloodstream, and intra-vascular stent, with a colonic perforation, allowed successful placement of a covered stent across the bleeding pseudoaneurysm and control of the source of bleeding, that would have likely led to the patient’s accelerated death from exsanguination. The patient recovered well from the bleeding after control of the source and, surprisingly, no evidence of bloodstream infection was there following this procedure (under antibiotic coverage).

Although in this instance it did not add significantly to the patient’s life span, this technique may be useful in other settings where the immediate survival of the patient relies on controlling the source of bleeding until definitive treatment (e.g. surgical bypass) can be pursued, or in the palliative setting when surgery is not an option.

## Data Availability

Data availability statement is not applicable.
